# Three Dimensional Collagen Scaffold Promotes Intrinsic Vascularisation for Tissue Engineering Applications

**DOI:** 10.1371/journal.pone.0149799

**Published:** 2016-02-22

**Authors:** Elsa C. Chan, Shyh-Ming Kuo, Anne M. Kong, Wayne A. Morrison, Gregory J. Dusting, Geraldine M. Mitchell, Shiang Y. Lim, Guei-Sheung Liu

**Affiliations:** 1 Centre for Eye Research Australia, Royal Victorian Eye and Ear Hospital, East Melbourne, Victoria, Australia; 2 Ophthalmology, Department of Surgery, University of Melbourne, East Melbourne, Victoria, Australia; 3 Department of Biomedical Engineering, I-Shou University, Kaohsiung, Taiwan; 4 O’Brien Institute Department, St Vincent’s Institute of Medical Research, Fitzroy, Victoria, Australia; 5 Department of Surgery, University of Melbourne, St Vincent’s Hospital Melbourne, Fitzroy, Victoria, Australia; 6 Faculty of Health Sciences, Australian Catholic University, Fitzroy, Victoria, Australia; Chang Gung University, TAIWAN

## Abstract

Here, we describe a porous 3-dimensional collagen scaffold material that supports capillary formation *in vitro*, and promotes vascularization when implanted *in vivo*. Collagen scaffolds were synthesized from type I bovine collagen and have a uniform pore size of 80 μm. *In vitro*, scaffolds seeded with primary human microvascular endothelial cells suspended in human fibrin gel formed CD31 positive capillary-like structures with clear lumens. *In vivo*, after subcutaneous implantation in mice, cell-free collagen scaffolds were vascularized by host neovessels, whilst a gradual degradation of the scaffold material occurred over 8 weeks. Collagen scaffolds, impregnated with human fibrinogen gel, were implanted subcutaneously inside a chamber enclosing the femoral vessels in rats. Angiogenic sprouts from the femoral vessels invaded throughout the scaffolds and these degraded completely after 4 weeks. Vascular volume of the resulting constructs was greater than the vascular volume of constructs from chambers implanted with fibrinogen gel alone (42.7±5.0 μL in collagen scaffold *vs* 22.5±2.3 μL in fibrinogen gel alone; *p*<0.05, n = 7). In the same model, collagen scaffolds seeded with human adipose-derived stem cells (ASCs) produced greater increases in vascular volume than did cell-free collagen scaffolds (42.9±4.0 μL in collagen scaffold with human ASCs *vs* 25.7±1.9 μL in collagen scaffold alone; *p*<0.05, n = 4). In summary, these collagen scaffolds are biocompatible and could be used to grow more robust vascularized tissue engineering grafts with improved the survival of implanted cells. Such scaffolds could also be used as an assay model for studies on angiogenesis, 3-dimensional cell culture, and delivery of growth factors and cells *in vivo*.

## Introduction

Engineered tissues are emerging as a viable approach to address the scarce supply of heterologous donor organs available for transplantation. However, vascularization is one of the great challenges that tissue engineering faces in order to generate sizeable tissue and organ substitutes that contain living cells [1]. In vascularized tissues, it is estimated that cells must be located within 150–200 μm of the nearest capillary to survive and function optimally [2]. In addition to access to oxygen and nutrients, vascular networks are needed for the removal of carbon dioxide and other cellular waste products during metabolism [2]. Therefore, engineering functional tissues with clinically relevant thickness will require approaches to incorporate vascular networks.

Several strategies have been explored to enhance tissue vascularization, including controlled release of proangiogenic factors such as vascular endothelial growth factor (VEGF) and fibroblast growth factor-2 [3,4], but such approaches could be limited by the short half-life of proangiogenic factors. Recent studies have reported that by allowing vascular cells to form rudimentary microvascular networks on biologically-derived scaffolds *in vitro*, it is possible to achieve enhanced integration of the implanted graft with host vasculature [5–8]. This pre-vascularization strategy generated ‘perfusable’ constructs that might improve survival of implanted cells when transplanted *in vivo*.

Collagen is a major extracellular matrix (ECM) component that has been widely used for constructive remodelling to facilitate cell growth and differentiation. The widespread use of collagen across many clinical applications is due to its bio-inductive, and desirable mechanical and degradable properties [9,10]. Collagen-based biomaterials in various formats have been employed as cellular scaffolds for tissue repair and regeneration [11]. Scaffolds made of collagen can support physiological processes in the course of healing and provide a platform for the development of 3-dimensional (3D) functionally intact tissue constructs with vascular in-growth, a component which is indispensable for survival of scaffold implanted cells. Type I bovine collagen has been used to create porous biomaterials as a means for regenerating bladder tissues in an experimental model of bladder exstrophy [12,13] and is readily available. It is therefore a suitable base material to fabricate a porous collagen scaffold.

In this current study, we sought to determine whether the collagen scaffold material could support *in vitro* vascularization with human endothelial cells and facilitate extrinsic and intrinsic vascularization when implanted *in vivo*. We employed the murine subcutaneous implant model to characterize the time course of endogenous cell infiltration and degradation of the collagen scaffold *in vivo*. We then employed a more complex rat tissue engineering chamber model to assess the potential of the collagen scaffolds to promote intrinsic vascularization and as a bioactive cue for sustaining the proangiogenic paracrine activity of human adipose-derived stem cells (ASCs) *in vivo*.

## Materials and Methods

### Synthesis and characterization of collagen scaffolds

**Fig 1:**
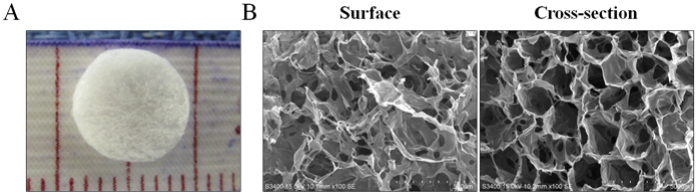
The 3D architecture and pore size of porous collagen scaffolds. (A) A 3D collagen scaffold measured to a diameter of 8 mm and a thickness of 2 mm. (B) Representative images captured by scanning electron microscopy on the surface (left) and through a cross section (right) of collagen scaffold.

The base collagen solutions were prepared as described previously [12]. Porous collagen scaffolds with an average pore size of 80 μm were generated from a lyophilized collagen solution (final concentration of 7 mg/mL). Briefly, 0.56 g of Type I bovine collagen isolated from cow skin was added to 80 mL of 0.5 M acetic acid solution (Merck Millipore, Darmstadt, Germany). The collagen solution was first frozen at -20°C for 24 hours, and then underwent a concentrated and lyophilized procedure to produce an interconnected porous structure. The pre-formed porous collagen scaffolds were then treated with N-Ethyl-N'-(3-dimethylamino-propyl) carbodiimide (Sigma-Aldrich, MO, USA), a crosslinking agent, for 4 hours and washed with phosphate buffered saline (PBS) three times. The scaffolds were then lyophilized again to improved mechanical strength (Table 1). Collagen scaffolds in the shape of a disc with a dimension of 8 mm diameter and 2 mm thickness (Fig 1A) were prepared for this study. The surface and cross-section morphology of the scaffolds were examined by a scanning electron microscope (Fig 1B).

**Table 1:** Mechanical properties of collagen scaffold.

Water content	97.6±0.3 (%)
Porosity	74.4±7.1 (%)
Young’s modulus	31.1±1.5 (10^6^gf/mm^2^)
Maximum elongation	3.4±0.2 (mm)

To assess the mechanical properties of the collagen sponge, the collagen sample was fully swollen in PBS solution before the mechanical strength measurements (tensile tests). In these tests we mounted the collagen onto the load cell using adhesive sponge gasket between the load cell and sample. The collagen membranes were cut into pieces (1 × 6 cm, n = 5) and the tensile strengths of the samples were measured up to the point at which they broke. The mechanical parameters of the samples were calculated and recorded automatically by using a material-testing system (MTS; Eden Prairie, USA) at a crosshead speed of 5 mm/min.

The porosity of the collagen sponge was determined using Archimedes’ principle [14]. The exterior volume (Vs) of the plug (1 cm × 1 cm) was measured using a Vernier caliper. The sample was then immersed in a pycnometer containing 99% ethanol solution. The actual volume (Va) of the sample was calculated using the formula:

Va=(Ww−Wo)−(Wt−Wp)0.789g/cm3

Where Ww is the weight of the ethanol and the pycnometer, Wo is the dry weight of the pycnometer, Wt is the combined weight of the ethanol, the pycnometer and the plug sample, Wp is the combined weight of the dry pycnometer and dry plug sample, and 0.789 g/cm^3^ is the density of 99% ethanol solution. The porosity of the plug was then determined using the following formula:

$$Porosity(\%)=\frac{Vs-Va}{Vs}100\%$$

Porosity values are expressed as means ± standard deviations (n = 5).

The water content (WC) of the collagen sponge was determined by swelling the sponge in a pH 7.4 PBS at room temperature. After the sponge was equilibrated with PBS, the wet sponge was blotted using filter paper to remove the water adherent to the sponge surface. The WC of the sponge was calculated as following formula:

$$WC(\%)=\frac{Ww-Wd}{Wd}100\%$$

Where Ww and Wd are the weights of the wet and dry sponge, respectively. The WC are expressed as means ± standard deviations (n = 5).

### Culture of human endothelial cells on collagen scaffolds and assessment of vascularization *in vitro*

Primary human dermal blood microvascular endothelial cells (hMECs) and growth media (EGM-2-MV) were purchased from Lonza (MD, USA). hMECs (5x10^5^ cells) were resuspended in 25 μL of human fibrinogen (15 mg/mL in PBS, Sigma-Aldrich) and dripped onto the upper surface of collagen scaffolds sitting flat on a petri dish. Following passive uptake of the cell/fibrinogen mixture, 25 μL of human thrombin (25 U/mL in PBS, Sigma-Aldrich) was added to form the fibrin gel. Scaffolds were then transferred to a 24-well plate containing 2 mL growth media and cultured for the specified time. Media was replaced after 2 days. At day 3, scaffolds were harvested and fixed in 4% paraformaldehyde for 24 hours before subjection to tissue processing and embedding in paraffin.

### Culture of human ASCs on collagen scaffolds *in vitro*

#### Isolation and culture of human ASCs

Human ASCs were isolated as previously described [15] following approval by St Vincent’s Hospital Human Ethics Committee (HREC5203). Fat tissues were collected as part of routine medical surgery and patients have given written informed consent for use of their tissues for research purpose. Briefly, aspirated fat tissues were washed with PBS containing 100 mg/mL streptomycin and 100 U/mL penicillin (Invitrogen, CA, USA), and minced into small pieces. Tissue fragments were washed once with PBS and then digested in 3 volumes of Dulbecco’s Modified Eagle's Medium (DMEM; Invitrogen) containing 0.5 mg/mL collagenase I (250 U/mg; Worthington Biochemical, NJ, USA) overnight with continuous shaking at 37°C. On the second day, cells were collected by centrifugation (800 x*g* for 10 minutes), seeded in culture flasks and cultured overnight at 37°C with 5% CO_2_ in growth media (DMEM supplemented with 10% fetal bovine serum (FBS, Sigma-Aldrich), 2 mM glutamine (Invitrogen), 100 mg/mL streptomycin and 100 U/mL penicillin (Invitrogen)). On the third day, attached cells were rinsed 3 times with PBS and cultured in growth media until confluent. Confluent cells were subcultured by trypsinization at a 1:4 ratio. We have confirmed that >95% of the isolated cells express mesenchymal surface markers CD29, CD44 and CD90 by flow cytometry [15].

#### Generating the GFP-expressed ASCs by lentiviral gene delivery

The packaging system for the production of lentiviral consisted of the following vectors: pCMV-ΔR8.91 plasmid, pMD.G (envelope element) and pLKO_AS2/GFP (transfer vector). These vectors were obtained from the National RNAi Core Facility (Taipei, Taiwan) as described previously [15]. Briefly, 6×10^5^ 293FT cells (Invitrogen) were seeded in a 6-cm culture dish and cultured for 24 hours. A mixture of the 3 vectors (2.5 μg of pCMV-ΔR8.91, 0.3 μg of pMD.G, and 2.5 μg of pLKO_AS2/GFP) was prepared and transfected into 293FT cells using Lipofectamine 2000 (Invitrogen). The virus-containing media were collected at 48 hours after transfection. For lentivirus transduction, human ASCs were seeded in 6-well plates (1×10^5^ per well) in growth media. After overnight incubation, cells were treated with virus-containing media containing 8 μg/mL polybrene (Sigma-Aldrich). The culture medium was removed at 24 hours post-infection and GFP positive cells were enriched (>99%) by FACSAria (BD Biosciences, CA, USA).

### Scanning electron microscopy (SEM)

Twenty-four hours after cell seeding, the scaffolds were directly subjected to SEM (Philips XL30 SEM, FEI Company, OR, USA) with an accelerating voltage of 24 kV at Melbourne Advanced Microscopy Facility (Parkville, VIC, Australia).

### Proliferation assay to assess cell growth *in vitro*

The growth of GFP-expressed human ASCs cultured in 3D collagen scaffolds or on collagen coated (0.5 mg/mL; Invitrogen) plate was assessed by using alamarBlue assay. Briefly, GFP-expressed human ASCs (1x10^6^ cells) were seeded in collagen scaffolds as 3D culture or in 12-well tissue culture plates as 2D culture, and cultured over a period of 3 weeks. At day 0, 2, 7, 14 and 21, scaffolds were cut into small pieces and incubated with alamarBlue® solution (Invitrogen) in a proportion of 1:10 (10 μL of alamarBlue® reagent to 100 μL of culture media) for 1 hour at 37°C in a humidified atmosphere containing 5% CO_2_. Fluorescence signals were measured at an excitation wavelength at 530–560 nm and an emission wavelength at 590 nm using a polarstar microplate reader (BMG Labtech, Australia) at 37°C.

### Animal use

Ethics approval for this work was obtained from the St Vincent’s Hospital Animal Ethics Committee (#021/13), in accordance with the requirements of the Australian National Health and Medical Research Council guidelines for the care and health of animals. The animals were supplied from Animal Resources Centre, Perth, Australia. No mice or rats became ill prior to the experimental endpoint. Minor stress induced by operation was looked for daily by observing the motility, grooming and feeding behaviour. All surgery was performed under general anaesthesia (2% isoflurane), and all efforts were made to minimize suffering. In the experimental endpoint, the mice or rats were euthanized using Lethobarb (200 mg/kg, intraperitoneal injection).

### Murine subcutaneous scaffold implantation to assess vascular infiltration *in vivo*

The collagen scaffolds filled with normal saline were implanted subcutaneously on either side of the dorsum of male C57B/L6 mice (10–12 weeks) under general anaesthesia (2% isoflurane). Scaffolds were then harvested at 1, 2, 4 and 8 weeks post-implantation to evaluate the time course of vascular infiltration and degradation of scaffold. At harvest, scaffolds were cleaned of connective tissues and immediately fixed in 4% paraformaldehyde overnight before being subjected to tissue processing and embedding in paraffin.

### Rat tissue engineering chamber to assess vascular infiltration *in vivo*

An *in vivo* rat tissue engineering chamber was employed as previously described [16]. Male Sprague Dawley rats weighing between 300–400 g were anesthetized with an inhalation of isoflurane. The femoral vessels were exposed through a longitudinal incision made on the medial thigh. Intact left and right femoral artery and vein were separated from each other and from the surrounding tissues over approximately a 2 cm distance and placed within an acrylic polymer chamber (internal dimensions of 10×8×4 mm; Department of Chemical and Biomolecular Engineering, University of Melbourne, Australia) by passing the intact vascular pedicle through openings at either end of the chamber, creating a flow-through model with an intact femoral circulation. A lid was attached to the chamber base to create a protected volume space for tissue growth. Each rat was implanted with two chambers (left and right femoral vessels); one containing a collagen scaffold impregnated with 100 μL of human fibrinogen gel (15 mg/mL; Sigma-Aldrich) and one with 100 μL of human fibrinogen gel alone to serve as a control.

To determine whether collagen scaffolds can be used to deliver cells with proangiogenic paracrine activity, scaffolds were seeded with human ASCs and implanted into the rat tissue engineering chambers. Male nude rats (CBH-rnu, 190–225 g) were purchased from the Animal Resources Centre (Perth, Australia) and were used for implantation of chambers containing either a collagen scaffold or a collagen scaffold pre-cultured for 48 hours with 1x10^6^ GFP-expressed human ASCs.

At 2–4 weeks post-implantation, tissue constructs were harvested from the animals under general anesthesia. Tissues were blotted dry and weighed. The volume of tissue constructs was determined by a volume displacement measurement method [17,18]. Tissues were fixed in 4% paraformaldehyde overnight. Tissue constructs were then divided into four equal transverse sections and paraffin embedded for routine histology and immunohistochemistry.

### Immunohistochemistry

Paraffin embedded sections (5 μm) were stained with eosin-hematoxylin (H&E) and Masson’s Trichrome to examine general tissue morphology. To identify hMECs in scaffolds cultured *in vitro*, endogenous peroxidase activity of sections was quenched with 3% H_2_O_2_ for 5 minutes. Sections were then subjected to enzymatic-mediated antigen retrieval with 0.1% Proteinase K (pH 7.8, Dako, Hamburg, Germany) for 8 minutes. The sections were blocked with 10% goat serum (Sigma-Aldrich) for 30 minutes, and then incubated with mouse anti-human CD31 (10.3 μg/mL, Dako) for 60 minutes. Sections were then incubated with biotinylated rabbit-anti-mouse secondary antibody (2.9 μg/mL; Dako) for 60 minutes and avidin-biotinylated-peroxidase complex (Vectastain Elite ABC kit, Vector Laboratories, Peterborough, UK) for 30 minutes.

To identify blood vessels in scaffolds implanted in mice, endogenous peroxidase activity of sections was quenched with 3% H_2_O_2_ for 10 minutes. Sections were then subjected to enzymatic-mediated antigen retrieval with 0.1% Proteinase K (pH 7.8, Dako, Hamburg, Germany) for 3 minutes. The sections were blocked with protein block solution (Dako) for 30 minutes, and then incubated with rat anti-mouse CD31 (3 μg/mL, BD Biosciences, North Ryde, Australia) for 60 minutes. Sections were then incubated with biotinylated rabbit anti-rat IgG (4 μg/mL, Dako) for 30 minutes, followed by avidin-biotinylated-peroxidase complex (Vectastain Elite ABC kit, Vector Laboratories).

To identify blood vessels in tissue constructs harvested from rat tissue engineering chambers, sections were treated with 3% H_2_O_2_ and proteinase K (Dako) prior to incubation with biotinylated Griffonia Simplicifolia lectin (6.67 μg/mL, B-1105, Vector Laboratories) overnight at 4°C. The incubation of lectin was then followed by HRP-streptavidin (1.78 μg/mL; Dako) treatment for 30 minutes.

To identify human cells, sections underwent heat-mediated antigen retrieval in citric acid buffer (pH 6.0, 30 minutes at 95°C) followed by 3% H_2_O_2_ for 5 minutes. Sections were then sequentially incubated in serum-free blocking solution (Thermo Fisher Scientific, MA, USA) for 10 minutes, rabbit anti-Ku80 antibody (0.06 μg/mL; Abcam, MA, USA) or mouse anti-human CD31 (4.1 μg/mL, Dako) overnight at 4°C, biotinylated goat-anti-rabbit secondary antibody (7.5 μg/mL; Vector Laboratories) or biotinylated rabbit-anti-mouse secondary antibody (2.9 μg/mL; Dako) for 60 minutes and avidin-biotinylated-peroxidase complex (Vectastain Elite ABC kit, Vector Laboratories) for 30 minutes. In all sections, peroxidase activity was visualized with diaminobenzidine chromogen (Dako) and sections were counterstained with hematoxylin before mounting in DPX mounting medium (VWR International, Poole, UK).

### Morphometric analysis of vascularization

Quantification of tissue vascularization and scaffold degradation were performed using video microscopy with a computer-assisted stereo investigator system (MBF Bioscience, VT, USA)[18,19]. For each sample, three cross-sectional sections—200 μm apart (scaffolds from *in vivo* murine subcutaneous implantation) or 4 complete transverse sections (tissue constructs from *in vivo* rat tissue engineering chambers) were counted with a 20x magnification objective. Using systematic random sampling, 12-point grids (400 μm x 400 μm) were superimposed on randomly selected fields representing 25% of the total area and each point within the grid was recorded as positive or negative on the basis of CD31 staining vessels, lectin staining vessels or scaffold. Percentage of vascular volume were determined by dividing the number of points in each of the selected fields that fell randomly on vessels (CD31 or lectin positive capillaries including the lumens) or scaffolds by the total number of points counted for that tissue section and multiplied by 100. Absolute vascular volume or scaffold of rat tissue was calculated by multiplying the percentage of vascular volume or percentage of scaffold material by the total tissue volume at harvest determined by volume displacement. All counting was completed by a trained operator masked to the identity of the tissue samples.

### Statistical analysis

Data are expressed as mean ± standard error of the mean (SEM). The mean data were analysed with unpaired t-test or one-way analysis of the variance (ANOVA) with Tukey multiple comparison post-hoc test where appropriate. A value of *p*<0.05 was regarded as statistically significant.

## Results

### Formation of capillary structures in collagen scaffolds seeded with human endothelial cells *in vitro*

**Fig 2:**
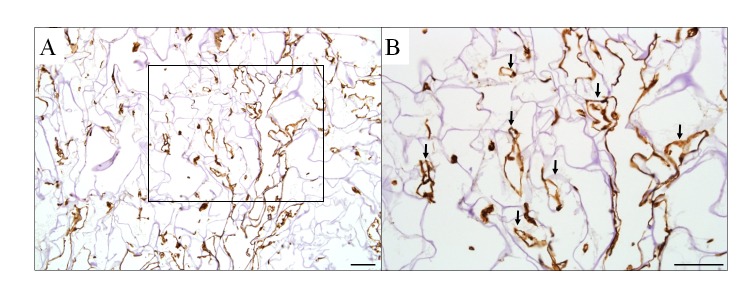
Capillary formation from human endothelial cells cultured in collagen scaffolds for 3 days. (A) Human dermal microvascular endothelial cells formed CD31^+^ capillaries with clear luminal structures at 3 days in culture. (B) A higher magnification of an inset box in (A) with capillary-like structures identified by arrows. Scale bar = 100 *μ*m.

To assess whether collagen scaffolds can provide a viable platform for the growth of endothelial cells, hMECs were cultured in fibrin filled scaffolds for 3 days. CD31^+^ capillary-like structures were formed throughout the scaffolds cultured with hMECs for 3 days (n = 2, Fig 2A and 2B).

### Collagen scaffold induces intrinsic vascularisation in a murine subcutaneous implantation model

**Fig 3:**
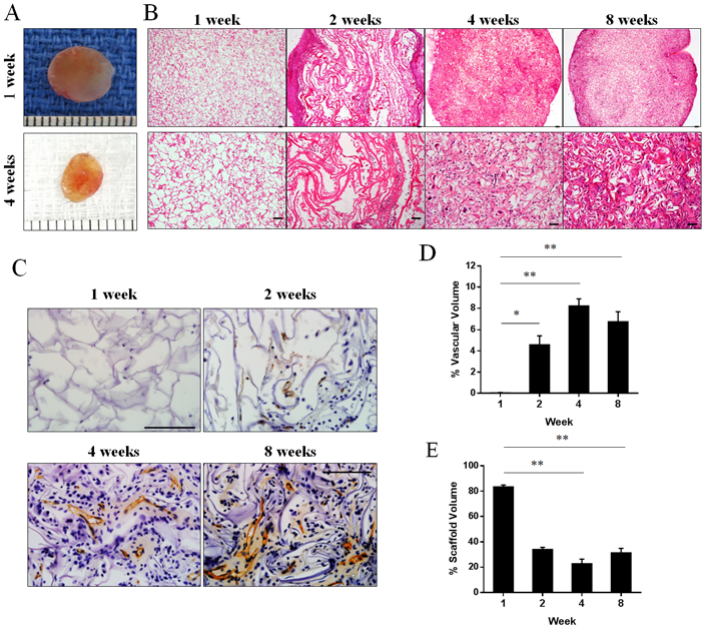
Vascular infiltration in collagen scaffolds implanted in mice. (A) Appearance of scaffolds harvested from mice at 1 and 4 weeks. Representative images of scaffold sections stained with haematoxylin and eosin (B) or endothelial cell marker CD31 (C) at 1, 2, 4 and 8 weeks post-implantation. Percentage of vascular volume (D) and percent of remaining collagen scaffold (E) at 1, 2, 4 and 8 weeks post-implantation (n = 3–6 mice). *p<0.05 and **p<0.01 by one-way ANOVA with Turkey post-hoc test. Scale bar = 100 *μ*m.

We assessed the degree of vascular infiltration of scaffolds harvested from mice at 1, 2, 4 and 8 weeks post-implantation (n = 4–6 from each time point). The scaffolds reduced in size the longer the implantation period (Fig 3A). Histological sections of scaffolds implanted for 1 week showed evenly distributed pores with minimal cell infiltration. The number of infiltrated cells gradually increased with longer implantation periods (2–8 weeks; Fig 3B). Within the scaffolds, CD31^+^ capillary-like structures with clear lumens were observed at 2, 4 and 8 weeks but not at 1 week (Fig 3C). Quantification of CD31^+^ vessels within the scaffolds confirmed that vascularization was minimal at 1 week but gradually increased from 2 weeks and reached a maximum percent vascular volume at 4 weeks (4.6±0.9% at 2 weeks, 8.2±0.7% at 4 weeks, 6.7±0.9% at 8 weeks; *p*<0.05 for each comparison vs. 0.04±0.04% at 1 week, n = 3–6; Fig 3D). The increase in CD31^+^ cells in the scaffold was accompanied by gradual degradation of scaffold material over time (22.7±3.7% at 4 weeks, 31.3±3.5% at 8 weeks; *p*<0.05 for each comparison *vs* 83.4±1.5% at 1 week, n = 3–6; Fig 3E). The extent of scaffold degradation plateaued at 4 weeks and collagen scaffold material could still be found at 8 weeks post-implantation.

### Collagen scaffold induces intrinsic vascularisation in a rat tissue engineering model

**Fig 4:**
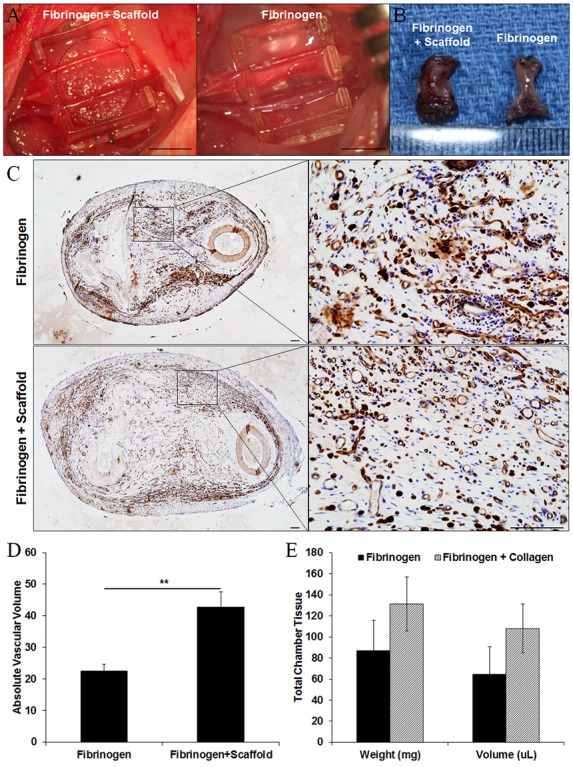
Tissue weight, volume and vascularity in tissue constructs generated in rat tissue engineering chambers. (A) The polyacrylic chambers containing collagen scaffolds infiltrated with fibrinogen gel (circled) or fibrinogen gel alone were placed around the femoral artery and vein in the groin region of rats. Scale bar = 5 mm. (B) Representative photomicrograph of new tissues generated in the tissue engineering chambers contained collagen scaffold infiltrated with fibrinogen gel (left) or fibrinogen gel alone (right) at 4 weeks post-implantation. (C) Representative images of tissue sections stained with blood vessel marker lectin. Top two figures: fibrinogen alone (-scaffold), bottom two figures collagen scaffold with fibrinogen (+scaffold). (D) The absolute vascular volume in vascularized tissues at 4 weeks post-implantation (n = 7). (E) The weight and volume of tissue constructs harvested at 4 weeks post-implantation (n = 7). **p<0.01 by unpaired t-test. Scale bars = 100 *μ*m.

An *in vivo* rat tissue engineering chamber was employed to assess the ability of collagen scaffolds to support an angiogenic response and new tissue formation (Fig 4A and 4B). Newly formed blood vessels were identified by lectin staining (Fig 4C). Compared to control chambers which contained fibrinogen gel alone, the absolute vascular volume was significantly higher in constructs generated from chambers containing collagen scaffolds infiltrated with fibrinogen gel (42.7±5.0 μL in collagen scaffold *vs* 22.5±2.3 μL in control; *p*<0.05, n = 7; Fig 4D). The percentage of vascular volume was similar between groups (40.7±2.5% in collagen scaffold *vs* 39.9±4.1% in control; *p*>0.05, n = 7). No significant difference in total chamber tissue weight and volume was found between groups (Fig 4E) and collagen scaffolds were completely degraded by 4 weeks post-implantation.

### Collagen scaffolds provide a bioactive cue for human ASCs *in vitro* and *in vivo*

**Fig 5:**
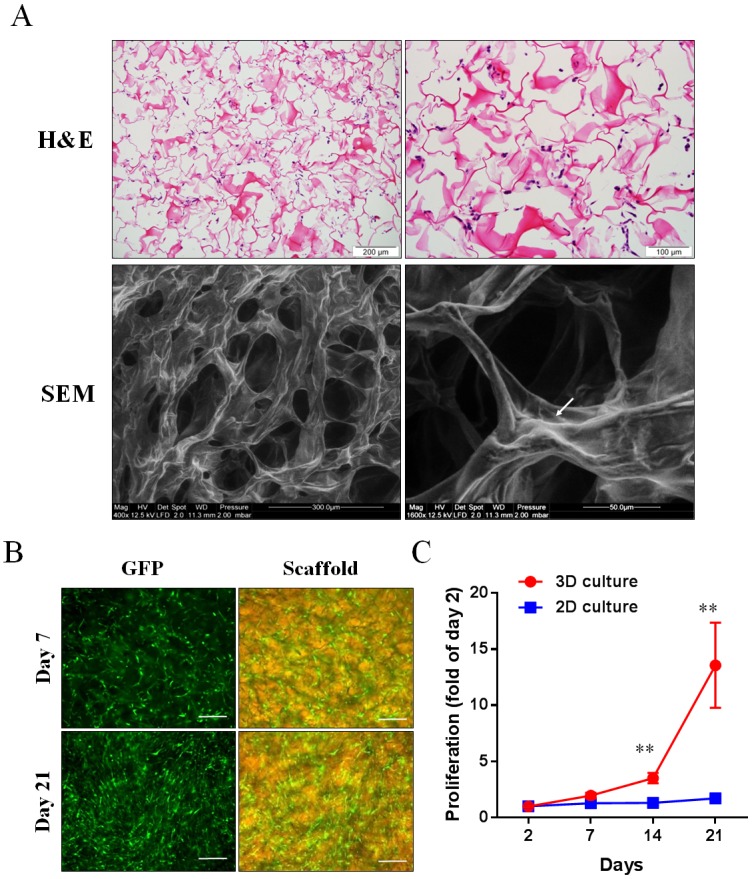
Morphology and growth of human ASCs cultured in 3D collagen scaffolds over 21 days. (A) Representative images captured from 3D collagen scaffolds seeded with human ASCs for 24 hours: haematoxylin and eosin (H&E) staining and scanning electron microscopy (SEM). The white arrow demonstrates human ASCs attached to the collagen scaffold. (B) Representative images of GFP-expressed human ASCs cultured in collagen scaffolds for 7 and 21 days. Scale bar = 200 μm. (C) The proliferation rate of GFP-expressed human ASCs cultured in 3D collagen scaffolds and on 2D collagen-coated plates (n = 3). **p<0.01 *vs* 2D culture by unpaired t-test.

Twenty-four hours after seeded onto the collagen scaffold, human ASCs were found distributed evenly throughout the collagen scaffold (Fig 5A). SEM showed that adhered human ASCs exhibited spindle shape morphology, and cell-cell and cell-scaffold interactions were observed around the porous structure of the collagen scaffolds (Fig 5A). Moreover, the proliferation of proangiogenic human ASCs [20] cultured in 3D collagen scaffolds and 2D culture was assessed over a period of 21 days. Those ASCs cultured in 2D (1x10^6^ cells/well in 12-well plate) were confluent at 2 days after seeding but did not proliferate further for the next 21 days due to contact inhibition. In contrast, human ASCs cultured in 3D collagen scaffolds continued to proliferate throughout the 21 days in culture (Fig 5B and 5C).

**Fig 6:**
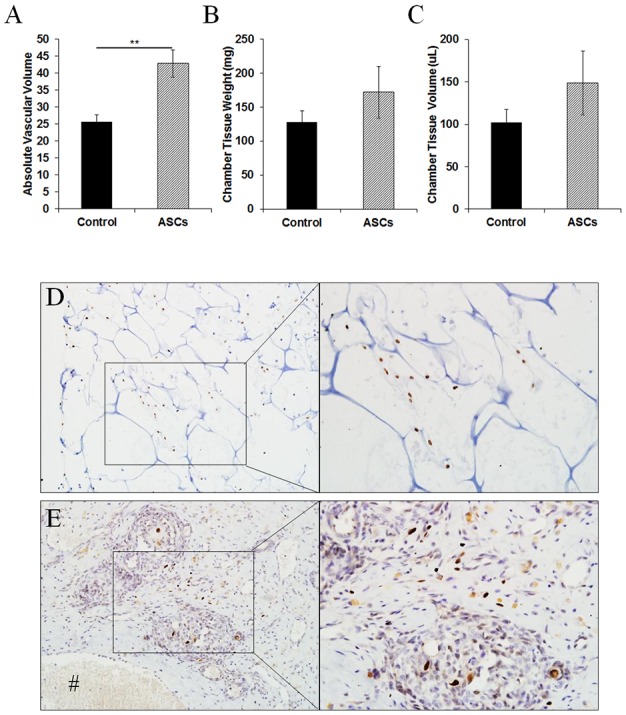
Tissue constructs engineered in rat tissue engineering chambers containing collagen scaffolds seeded with human ASCs. (A) The absolute vascular volume in tissue constructs harvested at 2 weeks post-implantation (n = 4). The total weight (B) and volume (C) of chamber tissue constructs harvested at 2 weeks post-implantation (n = 4). Human ASCs were identified by human-specific Ku80 (brown nucleus staining) in (D) the collagen scaffold and (E) the tissue construct harvested at 2 weeks post-implantation. **p<0.01 by unpaired t-test.

To determine whether collagen scaffolds support the proangiogenic function of GFP-labelled human ASCs *in vivo*, the formation of new vascularized tissue in the rat tissue engineering chambers was compared between chambers containing scaffolds seeded with and without GFP-labelled human ASCs. At 2 weeks post-implantation, the absolute vascular volume was significantly higher in the ASCs group compared to control group (42.9±4.0 μL in the collagen scaffold with human ASCs *vs* 25.7±1.9 μL in collagen scaffold alone; *p*<0.05, n = 4; Fig 6A). The percent vascular volume was increased in the ASC/scaffold group over the control scaffold group (32.1±5.0% in the collagen scaffold with human ASCs *vs* 25.9±1.7% in collagen scaffold alone; *p*>0.05, n = 4). No significant difference in total chamber tissue weight and volume was found between groups (Fig 6B and 6C). Staining with human-specific Ku80 showed that human ASCs were still present in the residual collagen scaffolds at 2 weeks post-implantation (Fig 6D) but human cells had also migrated into the neotissues outside the scaffold aligning close to the new blood vessels (Fig 6E). However, no human-specific CD31 positive cells were detected in the tissue constructs (data not shown).

## Discussion

The present study demonstrated that the 3D collagen scaffolds can support extrinsic and intrinsic vascularization using two different *in vivo* animal models, the murine subcutaneous implant model (extrinsic vascularization) and the rat tissue engineering chamber model (intrinsic vascularization). The collagen scaffolds support endothelial cell survival and permitted significant capillary formation within their pores *in vitro*. Furthermore, the collagen scaffolds also provide structural and mechanical support for 3D cell culture *in vitro* and can be employed as a bioactive cue for the delivery of human cells *in vivo*.

Collagen is one of the major ECM proteins that interacts with various growth factors and cells, and has a chemotactic activity to endothelial cells [21] in the wound healing process [22]. Theoretically, collagen scaffolds can be employed as cell carriers and as reservoirs for growth factors without compromising their activity which would be of great value to promote angiogenesis and vascular stabilization for tissue engineering [23,24]. Indeed, we have previously shown that exogenously added neuron cancer stem cells start to adhere to the scaffolds at day 1 and form a colony at day 3 [25]. In the present study, we have further demonstrated the cell compatibility of these collagen scaffolds with human endothelial cells and human ASCs. The human endothelial cells can be grown in the collagen scaffold and formed CD31^+^ capillary-like structures *in vitro* suggesting that the scaffolds can form a pre-vascularized platform for cell-based tissue growth for subsequent implantation. We also showed a greater growth of human ASCs when cultured in the porous 3D collagen scaffolds rather than the conventional 2D cell culture format. This was attributed to the interconnection of pores within the scaffolds providing more space to accommodate cell growth and allowing diffusion of nutrient, oxygen and metabolites through the scaffolds. Therefore, the collagen scaffolds provide a biological ECM microenvironment that supports growth of several human cell types including endothelial cells, ASCs and neuronal cancer stem cells [25].

The collagen scaffolds employed in the present study might well provide a bioactive cue for infiltrating cells, including endogenous endothelial cells, to induce vascularization of the implants. The porous structure of the collagen scaffolds allows the diffusion of nutrients as well as migration of cells [24], which are important features for vascularization. Xiao *et al*. [26] have recently shown that endothelial cells tend to accumulate around the pore interconnection of porous scaffolds, and it is the site where the cells migrate into the inner part of the scaffolds to induce vascularization. As such, the porous structure of scaffolds facilitates vascularization, and the collagen scaffolds are biocompatible when implanted *in vivo* in small animals. The murine subcutaneous implant model is often used to study the biocompatibility of scaffolds [27] as well as angiogenesis during wound healing [28]. An infiltration of host cells into the collagen scaffolds was seen at week 1 and an accumulation of host cells at the central region occurred from week 2. This time-dependent increased in cell infiltration correlated with an increasing number of CD31^+^ capillaries indicating vascularization of the implanted scaffolds. The gradual increased in vascularization of the implanted scaffolds was also found to be accompanied by the degradation of scaffold matrix over 8 weeks without adverse inflammatory response. These findings suggest that such scaffolds can integrate with the host tissues and provide a biodegradable environment for *in vivo* vascularization during a wound healing response [24].

Fibrin gel has often been used as a hydrogel in many applications of tissue engineering [29] such as skeletal muscle tissue [30] and cardiac tissue [31,32]. Fibrin gel can be derived from human fibrinogen and can potentially be applied clinically. Autologous fibrin gel can be synthesized from the patient’s own blood to minimise immune reaction [29] and this makes fibrin gel an attractive hydrogel for tissue repair and regeneration. Moreover, fibrin, an active component of the fibrinogen gel, has also been shown to be a pro-angiogenic factor that can enhance angiogenesis via triggers the angiogenic such as VEGF [33,34]. However, fibrin gel has poor mechanical properties render it susceptible to cell-mediated contraction and mechanical strain when implanted *in vivo* [35]. Brougham *et al*. [35] recently addressed this problem by incorporating fibrin into a collagen-glycosaminoglycan matrix hence reducing the degree of cell-mediated contraction as well as improving the viability of vascular smooth muscle cells. Similarly, we showed that incorporating fibrin gel into the collagen scaffolds yielded a greater vascular volume in the tissue constructs generated in an *in vivo* tissue engineering chamber compared with fibrin gel alone. The increase in vascular volume was likely due to larger chamber tissue volume of the constructs from the collagen scaffold group as the percentage of vascular volume was similar between groups. Thus, the structural support provided by the collagen scaffolds would synergise the growth of tissues in fibrin gel.

An alternative approach to using soluble growth factors to promote intrinsic vascularization would be seeding scaffolds with pro-angiogenic cells [5–8]. Human ASCs have been known to release a variety of pro-angiogenic cytokines and growth factors including interleukins, VEGF, angiogenin, and fibroblast growth factor-2 under normoxic [36] and hypoxic [37] conditions. The level of cytokines and growth factors released from human ASCs has been found to be sufficient to promote proliferation of human endothelial cells [36,37] and promote angiogenesis *in vivo* [36]. Moreover, Wang *et al*. [20] recently developed a biocompatible polytetrfluoroethylene filtration membrane to study the pro-angiogenic activity of human ASCs. This device was used to encapsulate human ASCs to insulate the implanted cells from the host microenvironment while allowing diffusion of soluble factors secreted from the cells. When implanted subcutaneously in rats, ASC-laden devices resulted in higher number of blood vessels in the tissue surrounding the devices and expressed higher levels of cytokines and growth factors such as interleukin-10 and VEGF in comparison to acellular devices [20], confirming a pro-angiogenic effect of human ASCs *in vivo*. We found that ASC-laden collagen scaffolds resulted in a higher volume of blood vessels in tissue constructs in comparison to scaffolds without ASCs. At 2 weeks post-implantation, some implanted human ASCs remained in the collagen scaffolds while others have migrated out of the scaffolds into the neotissues and localized in close proximity to the host blood vessels. However, immunostaining with human-specific endothelial marker CD31 did not detect positive cells in the tissue constructs suggesting that the increase in vascular volume was likely attributed to the paracrine activity of the implanted human ASCs on host tissues, rather than the differentiation of ASCs into endothelial cells.

## Conclusions

We have demonstrated a 3D porous collagen scaffold material that is versatile, biocompatible, biodegradable and has bioactivity that mimics the functions of natural ECM to support vascularization *in vitro* and *in vivo* in different species (rat and mouse) and experimental models (subcutaneous implant and tissue engineering chamber). Unique properties of the collagen scaffolds which (1) provide structural support for cell infiltration and angiogenesis, (2) can be used as a cell carrier for *in vivo* implantation, and (3) offer mechanical support to grow more robust vascularized tissue grafts for tissue and organ repair.
